# Direct observations of American eels migrating across the continental shelf to the Sargasso Sea

**DOI:** 10.1038/ncomms9705

**Published:** 2015-10-27

**Authors:** Mélanie Béguer-Pon, Martin Castonguay, Shiliang Shan, José Benchetrit, Julian J. Dodson

**Affiliations:** 1 Département de Biologie, Université Laval, Pavillon Vachon, 1045 Avenue de la Médecine, Université Laval, Québec, Québec, Canada G1V OA6; 2 Department of Oceanography, Dalhousie University, 1355 Oxford Street, PO Box 15000, Halifax, Nova Scotia, Canada B3H 4R2; 3 Institut Maurice-Lamontagne, Pêches et Océans Canada, 850 Route de la Mer, C.P. 1000, Mont-Joli, Québec, Canada G5H 3Z4

## Abstract

Since inferring spawning areas from larval distributions in the Sargasso Sea a century ago, the oceanic migration of adult American eels has remained a mystery. No adult eel has ever been observed migrating in the open ocean or in the spawning area. Here, we track movements of maturing eels equipped with pop-up satellite archival tags from the Scotian Shelf (Canada) into the open ocean, with one individual migrating 2,400 km to the northern limit of the spawning site in the Sargasso Sea. The reconstructed routes suggest a migration in two phases: one over the continental shelf and along its edge in shallow waters; the second in deeper waters straight south towards the spawning area. This study is the first direct evidence of adult *Anguilla* migrating to the Sargasso Sea and represents an important step forward in the understanding of routes and migratory cues.

## Abstract (web summary)

Migration of adult American eels from the coast to their spawning area in the Sargasso Sea have previously only been inferred from larval distributions. Here, Béguer-Pon *et al*. track adult eels from the continental shelf into the open ocean, with one individual migrating to the northern limit of the spawning site.

## Graphical abstract

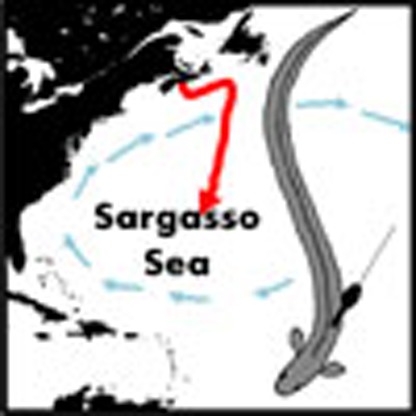

At the onset of maturation, American eels (*Anguilla rostrata*) undertake a long-distance migration (up to 4,000 km) from continental waters to their single spawning area in the Sargasso Sea as inferred from the collection of leptocephalus larvae during several marine expeditions that began in 1904 (refs 1, 2). After over a century of research that has failed to catch a single adult in the open ocean, most information concerning their spawning migration remains a complete mystery. Neither the exact location of the spawning site nor the migration routes and the environmental conditions along these routes are known. The American eel is listed as endangered on the International Union for Conservation of Nature (IUCN) red list^3^ and assessed as threatened in Canada^4^. Considering the species' precarious status, filling the knowledge gap with respect to its marine migration is important for both research and management objectives. More specifically, this implies refining information on the extent of the spawning area, describing the environmental conditions experienced during the migration and revealing the migration routes and orientation cues eels use. Acoustic tracking of American eels in the St. Lawrence River and Estuary revealed that maturing (silver) eels must use selective tidal stream transport to successfully leave the Estuary^5^. In 2011, attempts to track silver American eels in marine waters using pop-up satellite archival tags (PSATs) began in the Gulf of St. Lawrence (Canada)^6^. However, a high rate of predation of tagged eels by homeothermic fishes prevented successful tracking of the eels beyond the Gulf. This same technology was used to track European eels (*A. anguilla*) at sea, and that study provided unique behavioural insights into the early phase of their marine migration^7^,^8^.

In an attempt to follow American eels *en route* to their spawning site and thus document the full-marine migration, a total of 38 silver eels were tracked with PSATs. Two types of PSATs were used, they are: 27 X-tags from Microwave Telemetry (http://www.microwavetelemetry.com) and 11 SeaTag-GEOs from Desert Star Company (http://desertstar.com/). The tagged eels were released on the Scotian Shelf, off Nova Scotia (NS), Canada, in the fall of 2012, 2013 and 2014. While eels tagged in 2012 and 2013 were from NS rivers, those released in 2014 were captured in the St. Lawrence Estuary (SLE) and transported by truck to NS (migration shortcut of 1,400 km). The latter approach was used given the considerably larger size and body mass of eels from the SLE relative to NS-native eels (2.9 kg on average versus 1.4 kg), thereby reducing the potential negative impact of carrying the PSAT. To document the horizontal and vertical behaviours of the migrating eels, we analysed data from 28 PSATs that successfully transmitted their data. Daily trajectories were reconstructed by matching the environmental data recorded by the tags (water temperature and either depth or geomagnetic field total intensity) with outputs of the operational global ocean circulation models that have advanced data assimilation components. As satellite tags were unable to collect measurable light intensity because of the propensity of eels to avoid the euphotic zone during daytime, reliable sunset and sunrise estimates were not available, preventing us from inferring the longitude as traditionally performed by other large-scale tracking studies of marine species^9^. Nevertheless, we were able to infer ranges of longitude in certain cases^10^, using sunrise and sunset times estimated from marked diel vertical migration (DVM) for some days and some individuals.

Eight eels were successfully tracked to the open ocean off the continental shelf, including one tracked for 2,400 km to the northern limit of the spawning site in the Sargasso Sea. Our results represent the first direct evidence of adult *Anguilla* migrating to the Sargasso Sea. The similarity of trajectories and behaviour of migrating eels indicate a degree of consistency in the orientation/navigation mechanism employed throughout the migration. The migratory routes seem to be largely independent of current fields. Although migrating mainly in shallow water over the continental shelf with no marked vertical migratory pattern, eels exhibit DVM down to 700 m in the open ocean.

## Results

### Pop-off location and tracking success

**Figure 1:**
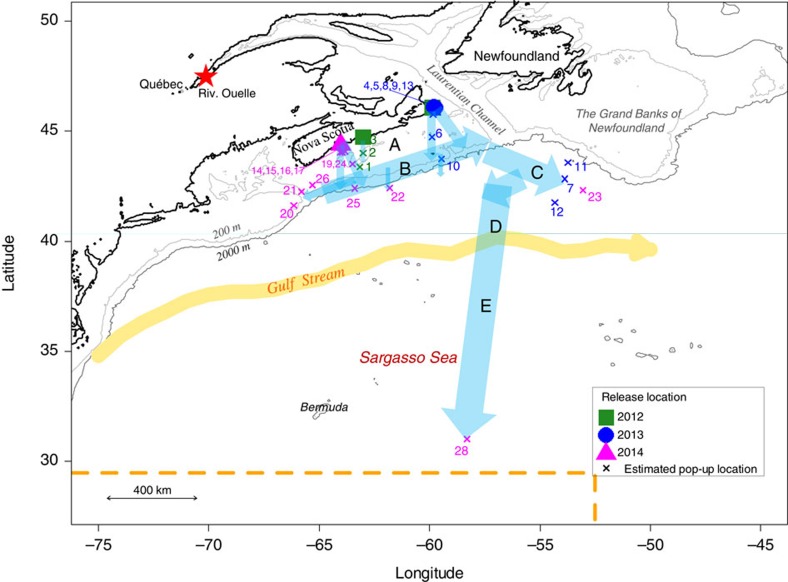
Release locations of eels equipped with PSAT, their pop-up locations and a schematic of the main reconstructed headings (blue arrows). A is the Scotian Shelf, B represents the edge of the Scotian Shelf, C represents the exit of the Laurentian Channel (open ocean), D represents the area after C, which includes the Gulf Stream and E the Sargasso Sea. The orange arrow represents the north wall of the Gulf Stream in November. The red star represents the capture location of the eels used in the 2014 experiments. Eels of other years were captured nearby their release location. The dashed orange line represents the northern and eastern limits of the American eel spawning site inferred from the collection of leptocephalus larvae^11^. Each number represents a satellite tag.

Of the 38 PSATs attached to eels and released on the Scotian Shelf over 3 years, 28 successfully transmitted their data after popping off at an estimated distance of 5–1,570 km from their release site (Fig. 1 and Supplementary Table 1). The rate of non-reporting was particularly high in 2012 when 70% of the X-tags (7/10) never transmitted to satellites compared with 17% in 2013 (1 SeaTag-GEO) and 6% in 2014 (1 X-tag; Supplementary Table 1). The 18 reporting X-tags transmitted on average 97% (range: 56–100%) of their archival data, whereas the average transmitting rate for the 8 SeaTag-GEOs was 48% (range: 7–100%). The PSATs transmitted their data 24.4 days on average after their deployment (range: 0.7–59.7 days), that is, long before their scheduled transmission date (which was 3–5 months after deployment). Reasons for these premature releases were unclear except for two predation events clearly identified (see below). A total of 431 days of eel tracks were obtained, with the longest track reaching 57.2 days and the shortest only 0.5 day (Supplementary Table 2). Six eels were tracked for more than a month (31.8–57.2 days), eight eels were tracked between 12.7 and 28.7 days and the remaining ten eels were tracked for less than 8 days.

**Figure 2:**
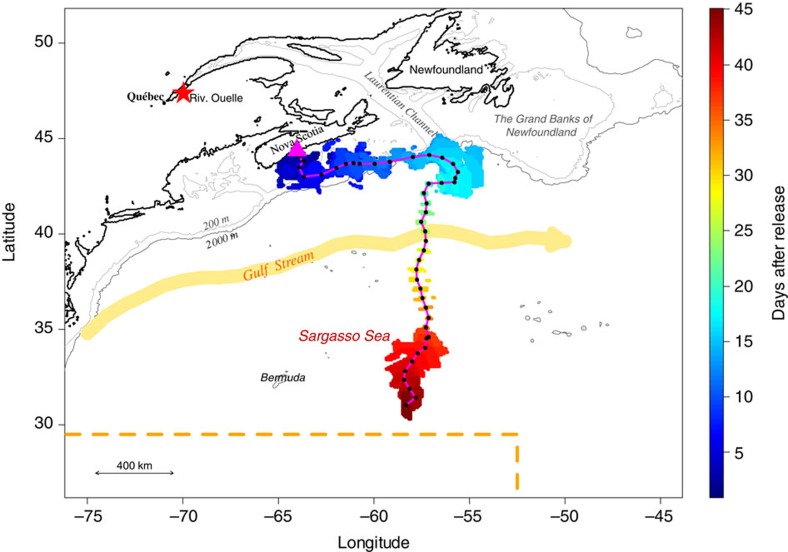
Reconstructed daily locations of the eel #28 equipped with X-tag #141105. The red star represents the capture location of this eel in the SLE while it was performing its downstream migration. The magenta triangle represents the release location after tagging. The black dots are the mean reconstructed daily locations and the magenta line is the corresponding mean trajectory. A colour gradient is used to show the temporal dimension of the reconstructed trajectory (each day is represented by a colour). The range of potential daily locations is lowest for days 19–33.

The pop-up locations were estimated to be beyond the Scotian Shelf for five of the eels, at a great-circle distance (that is, the shortest distance between two points on the surface of a sphere) of 555–1,570 km from the release site (2 X-tags and 3 SeaTag-GEOs, Fig. 1 and Supplementary Table 2). The uncertainty around these estimated locations were on average 120±94 km in latitude (mean±s.d.; range: 9–288) and 77±37 km in longitude (range: 20–148; Supplementary Table 3). The estimated pop-up locations were generally relatively close to the first transmitting location estimated from Argos. For instance, the PSAT that went the furthest began transmitting its data at 32.37°N, 58.26°W and the reconstructed pop-up location was estimated to be 30.33–31.67°N, 58.08–58.50°W (Figs 1 and 2, and Supplementary Tables 1 and 2), which is very close to the northern limit of the spawning area^2^. This provides the first direct evidence of American eel migrating from coastal waters to the Sargasso Sea spawning site. The pop-up location of six PSATs was estimated to be beyond (or very close to) the edge of the Scotian Shelf, at a distance of between 230 and 354 km from their coastal release sites, whereas the remaining PSATs (17) remained relatively close to their respective release sites (a few km to 111 km).

### Reconstructed paths and behaviour

The migratory paths were reconstructed for 16 eels equipped with X-tags and 4 eels equipped with SeaTag-GEOs (Supplementary Tables 2 and 3). The uncertainty of reconstructed locations varied considerably among tags and days, averaging 120±91 km in latitude and 207±170 km in longitude for X-tags and 206±127 km of latitude and 320±201 km in longitude for SeaTags-GEOs (Supplementary Table 3). In some cases, the uncertainty of the daily reconstructed locations was as little as 9 km of latitude and 7 km of longitude (for example, eel #28 (in the open ocean) equipped with an X-tag), allowing the reconstruction of relatively precise trajectories. However, uncertainty was generally quite high along the shelf break front of the Scotian Shelf (area B, Fig. 1), reaching several hundred kilometres in longitude. We were thus unable to evaluate potential bidirectional movements (southwestward or northeastward) at the shelf edge for many eels.

**Figure 3:**
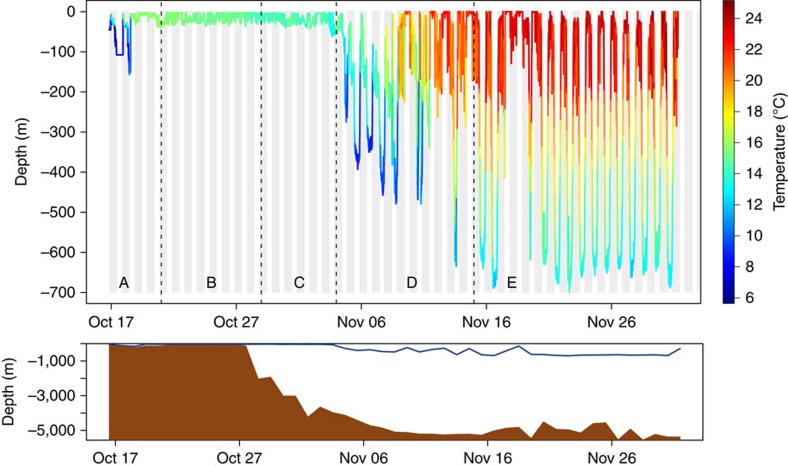
Vertical behaviour of eel #28 equipped with the X-tag #141105 along its journey from the Scotian Shelf to the Sargasso Sea. The temperature experienced along the way is superimposed to the depth profile. The bottom panel shows the observed daily maximum eel depth (dark blue line) and the corresponding bottom depth at the daily reconstructed location (brown-shaded curve). A is the Scotian Shelf, B represents the edge of the Scotian Shelf, C represents the exit of the Laurentian Channel (open ocean), D represents the area after C, which includes the Gulf Stream and E represents the Sargasso Sea.

The reconstructed paths indicate a migration distance between ca 1,300 and 1,700 km for four eels and of ca 2,400 km for the eel that reached the Sargasso Sea (Supplementary Table 2 and Fig. 2). The inferred trajectories and behaviour of eels within five different areas are summarized in Table 1 and Fig. 1 (also see Supplementary Table 2). Despite differences in release years, locations and eel origins, similar paths and vertical behaviours were observed. Following their release over the Scotian Shelf (area A), all eels immediately headed south and slightly to the east towards the edge of the continental shelf (Fig. 1). During this first stage of the migration, lasting between 3 and 13 days (7.2 days on average, *N*=11), eels equipped with X-tags generally exhibited DVM, occupying shallow waters at night (<50 m) and bottom waters during the day (maximum of 240 m in the Emerald Basin on the Scotian Shelf, see bathymetry in Supplementary Fig. 1). Eels experienced a positive gradient of temperature while travelling from the coast to the edge of the Scotian Shelf:*+*ca 8 °C in ca 200 km from the southernmost release location (Supplementary Figs 2–4). Once over the edge of the Scotian Shelf (area B), they remained in shallow waters, performing between 5 and 23 dives per day without any obvious diel pattern, and lasting from 4 to 26 days for individuals tracked beyond that area. Although it was not possible to assess the trajectories in that area because of the relatively high uncertainty of daily locations for five eels, seven eels were shown to have headed northeastward, swimming against the southwestward shelf-break current. Five of these eels then migrated to the southeast, reaching the exit of the Laurentian Channel (the so-called Laurentian Fan) and west of the Grand Banks off Newfoundland (area C), 17–57 days after their release (average of 36.4 days). Of those five eels, the smallest three which were equipped with SeaTag-Geo exhibited mean migration speeds relative to the ground between 10 and 17 km per day, whereas the two largest eels, equipped with X-tags, showed much higher speeds of 38 and 50 km per day. Eels again experienced a positive gradient in salinity (ca +3.2 in about 170 km) while travelling in this area (Supplementary Figs 2–4). The two eels equipped with X-tags (which record depth) exhibiting clearly discernable DVM between the surface and ca 400–500 m in depth once the inferred salinity was greater than 35 (Fig. 3 and Supplementary Figs 3–5).

The tracking continued for one eel (eel #28), which suddenly modified its trajectory and headed south while performing vertical migrations that were not clearly diel (area D; Fig. 2). It crossed the Gulf Stream 29 days after its release (Supplementary Fig. 3) and reached 45 days after release a latitude of 30.33–31.67°N, that is, 92–242 km from the 29.5°N northern limit of the spawning site^11^, at a mean travel speed of 49 km per day. It performed marked DVM, with an average depth of 141±14 m at night and 618±16 m during the day (maximum of 699 m), during its relatively direct southerly trajectory into the Sargasso Sea (area E, Fig. 3). Another eel (eel #10) reached the Gulf Stream 12 days after its release but failed to cross it, and instead was tracked to the edge of the Scotian Shelf several days later, indicating a reverse movement (Supplementary Fig. 6).

Eels experienced a very wide temperature range during their migration, from 2.5 °C over the Scotian Shelf to 25.1 °C in the Sargasso Sea (Supplementary Table 2).

### Predation events

Premature releases for two X-tags were clearly due to predation by homeothermic fishes. These predation events were identified from the sudden increase in ambient temperature recorded by the tags (Supplementary Fig. 7 a,b). The tags were also not able to record any light data during the predation periods. One eel (#18) was eaten while on the Scotian Shelf only 1.5 days after its release and the tag remained inside the predator's stomach for 6.7 days as identified by the high temperature (between 20.5 and 23.6 °C) during that period. The second eel (#27) was subject to predation after 34 days of activity and the tag remained in the predator's stomach for 3.3 days with observed temperatures varying between 19.2 and 27.1 °C. The depth recorded by the tag just before this predation event showed that it occurred beyond the Scotian Shelf (Supplementary Fig. 7 a,b). The statistical method previously used and detailed in our previous tracking study conducted in the Gulf of St. Lawrence^6^ was applied to identify the two predators. The predator of eel #18 was identified as a porbeagle shark (*Lamna nasus*), whereas the predator of eel #27 was most likely a Bluefin tuna (*Thunnus thynnus*). Potential predation events by ectothermic fish could also have occurred for seven of the eels equipped with X-tags, as suggested by their vertical migratory behaviours shortly after their release and over the Scotian Shelf. Indeed, these eels generally started to exhibit DVM immediately after their release for several days (1.5–7 days) before suddenly descending to the bottom (Supplementary Fig. 7c). They then remained for 1.5–14 days at the bottom (constant depth, zero light) before suddenly reaching the surface (the release mechanism was then triggered following 7 days at the surface).

## Discussion

This large-scale study has successfully reconstructed daily locations of migrating eels at sea and is the first to have tracked one individual from the coast to the northern limit of the spawning area in the Sargasso Sea. This study therefore represents an important step forward in the understanding of the oceanic migration of anguillid eels and the possible orientation mechanisms used by the species. The similarity of trajectories and behaviour of migrating eels in this study indicate a degree of consistency in the orientation/navigation mechanisms employed throughout the migration. Two distinct migratory phases are identified: one in shallow waters and the other in deep waters off the shelf. The first phase of the marine migration, from the coast to oceanic/deep waters (salinity <35), may simply rely on gradients and fronts associated with salinity and temperature. Both physical factors increase from the coast to open waters and could thus have guided eels towards the edge of the Scotian Shelf away from the coast immediately after their release. The eels then moved mainly eastward along the edge of the Scotian Shelf where they experienced relatively constant temperature and salinity. If the eels were seeking thermohaline gradients, it could explain their departure from the Scotian Shelf at the exit of the Laurentian Channel where the waters exiting the Gulf of St. Lawrence are colder and less saline. Migration along the edge of the continental shelf was also observed for European eels tracked in the North Sea^12^. In that study, all tagged eels but one headed north while on the Norwegian shelf. Assuming that eels were searching for thermohaline gradients, two opposite migration headings (north or south) were possible, such as in our present study over the Scotian Shelf (southwest or northeast). Although the reasons why most American eels headed northeast over the Scotian Shelf edge and European eels headed north over the Norwegian Trench are unknown, this does indicate that orientation cues other than those provided by thermohaline gradients are involved for that portion of the migration. Nevertheless, in both studies some eels were observed to have headed in the opposite direction, reinforcing the hypothesis that thermohaline fronts provide some orientation cues for migrating eels.

During the second phase of the marine migration (salinity >35, bottom >2,000 m), the eel tracked to the Sargasso Sea exhibited a relatively sudden change in direction, heading south to the northern limit of the spawning site in a quasi-straight line from the area adjacent to the exit of the Laurentian Channel. The orientation/navigation cues are unknown but there appears to be clear thermohaline variation for the first part of this second phase from the continental shelf to the southern edge of the Gulf Stream (Fig. 3); so thermohaline cues could be used for orientation up to that point. However, the speed and directionality of the last portion of the track in the Sargasso Sea for a fish that has never before experienced such a migratory trajectory in an ocean with only weak horizontal gradients suggests the involvement of an inherited bi-dimensional map similar to that proposed for Pacific salmon^13^. Indeed, the latter study showed that salmon use a combination of geomagnetic intensity and inclination angle to assess their geographic location. The sensitivity of eels to the geomagnetic field has been known for a long time^14^,^15^ and recent experiments by Durif *et al*.^16^ supported the conclusion that eels have a magnetic compass that they can use for orientation. The existence of a neural substrate for a vertebrate magnetic sense was also recently demonstrated^17^. It thus seems likely that eels do possess a magnetic map and true navigation abilities.

In the marine environment, several fish species undertake long-distance migrations and must possess equally impressive navigation abilities^18^. The Atlantic bluefin tuna (*Thunnus thynnus*), for instance, is one of the best documented cases in the Atlantic ocean, with transatlantic seasonal migrations between the Gulf of Mexico and the eastern Atlantic or Mediterannean sea (several thousands of kilometres) and strong interannual fidelity to spawning sites^19^. Another known example involves mature female porbeagle sharks (*Lamna nasus*), which migrate ∼2,400 km to a subtropical pupping ground in the Sargasso Sea^20^. Both fish species are known eel predators^6^,^21^,^22^.

The eel tracked into the Sargasso Sea was an eel translocated from the SLE to the Scotian Shelf. Its behaviour during the first phase of the marine migration was similar to the behaviour of other translocated and non-translocated eels (from previous years) suggesting that translocation had no discernable effects on the large-scale behavioural patterns documented here. In contrast, a recent study attempted to track translocated European eels *en route* to the Sargasso Sea by releasing them in the open ocean several thousand kilometres from their native continental waters^8^. The 19 translocated eels in that study exhibited a broad range of directions and no consistent migratory patterns were observed. However, eels used in the European experiment were artificially matured and kept in captivity for weeks or months before their release, probably interfering with the normal developmental process of migration and maturation. It is also possible that silver eels need cues from continental shelf waters to orientate across open oceanic waters. Translocated American eels used in our study were not artificially matured and were released in continental shelf waters within a few days of capture, thus minimizing the aforementioned problems. Given the relative success of the approach reported in the present study, additional experiments using similar protocols should be pursued to confirm the migratory patterns documented here.

Silver eel migratory routes seem to be largely independent of current fields. Instead of taking advantage of oceanic currents, they may travel against them as was observed for European eels tracked in the North Sea^12^. American eels leaving the Scotian Shelf must cross the Gulf Stream, a strong northeastward current. One of our tracked eels managed to do this and its track was little affected by this crossing even though a slight drift was observed along its path towards the spawning area. However, this eel was among the largest individuals that can be found in North America (2.8 kg, 113 cm) and it is likely that smaller eels tagged with PSAT would have trouble crossing the Gulf Stream as we observed for one eel released in 2013 (1.3 kg, 93 cm).

Our results indicate that DVM does not occur along the whole migratory path. Over the continental shelf and at its edge, most eels did not exhibit DVM and remained in shallow waters, performing multiple dives day and night. In that area, the horizontal salinity gradient is greater near the surface than in deeper waters (100–200 m). Remaining in shallow waters and periodically diving to greater depths may represent a mechanism by which eels sample the salinity gradient to obtain directional information leading offshore. Well-defined patterns of DVM appeared once the salinity was greater than 35 and remained constant to the northern limit of the spawning area. DVM behaviour has been observed among many anguillids in the early stages of their marine migration^7^,^23^,^24^ and some authors have speculated that such behaviour is a trade-off between predator avoidance and the necessity to maintain sufficiently high metabolism for migration. The American eel tracked to the Sargasso Sea showed a clear bimodal distribution in ambient temperatures such as seen in European eels directly released in the Sargasso Sea^8^. Such vertical behaviour observed in both American and European eels is consistent with the hypothesized trade-off between predator avoidance and the metabolic requirements of migration.

An important rate of non-reporting by tags and premature releases were two important issues encountered in this study, as in similar studies of eels tagged with PSAT. In our study, the overall loss rate of tags was 26.3%, which is approximately the same as other PSAT studies in eels (11.1–32% (refs 7, 8, 23, 24, 25, 26)) and close to the average from a review conducted on the performance of PSAT on other fish species (21% (ref. 27)). The reasons for non-reports are unknown but could be due to tag malfunctioning, destruction by predation or inability of the tag to transmit their data to satellites. In our study, the loss rate was particularly high during the 2012 experiment during which eels were released directly from the shore, in shallow waters, unlike in other years. The vertical behaviour of eels immediately after their release in deeper waters suggests that eels released on the shoreline may have taken refuge under rocks and in the substrate where they dislodged their tags. The non-reporting tags may have remained stuck in the substrate or under rocks, therefore preventing data transmission, or they may have washed up on the shore in a position preventing satellite transmission.

Releasing eels 5–10 km offshore over deeper waters appeared to be a more successful method. Nevertheless, premature release occurred for all tags. Such a high rate of premature release was also reported in similar tagging studies in which 50–92.8% of the PSATs started transmitting their data before the scheduled pop-up date. In our study, predation by warm-gutted fish was clearly responsible for the premature release of two tags. Premature release of the other tags could also have been caused by predation by ectothermic fish. For several tags that successfully transmitted their data, a sudden descent to the seabed was observed a few hours or days after their release. These tags remained for several consecutive days on the bottom (100–200 m) before suddenly rising to the surface definitively. These sudden changes in vertical behaviour as well as the absence of light data and the absence of temperature increases may be due to predation by ectothermic fish. Such potential predation events not accompanied by an increase in temperature were also reported in other studies that tracked eels using PSATs^8^,^12^. Finally, for the eels tracked the longest, their tags suddenly reached the surface terminating a continuous period of regular behaviour (DVM), such as in ref. 8, suggesting predation of the eel without the tag or more likely a failure of the attachment.

Although we have tracked eels with the best telemetry technology available, a certain degree of uncertainty concerning the observations reported here is associated with two limitations of the technology; the potential impact of the tags on the behaviour of fish of relatively small body mass and limited data retrieval from the satellite tags (see ‘Methods' for a more extensive discussion of potential drawbacks). Several laboratory studies have shown that PSATs increase drag and can significantly impair the swimming performance of relatively small eels^28^,^29^,^30^. This may have contributed to slower speeds of smaller eels and the more westerly pop-up locations on the Scotian Shelf. Although DVM has been reported for eels tagged with much smaller, internally-placed, acoustic tags, it remains unknown if the maximum and minimal depths at which eels swim could be affected by the external PSAT. Another drawback of using PSATs is associated with the limitations of retrieving recorded data and its impact on the reconstruction of migratory paths. For the X-tags, we used the depth data available at 15-min intervals to infer the longitude^10^. Because of the data sampling rate and the individual and daily variability of DVM^10^, the uncertainty in longitude estimates was around 1.2° (∼400 km). The latitudes were inferred using the temperature recorded at specific depths by X-tags. The uncertainty of the reconstructed latitudes thus comes from the accuracy of the recorded data and from the resolution and accuracy of the operational ocean circulation models used to compare with the archival data (7–9 km). This defines the minimal uncertainty of the reconstructed path and prevents assessment of finer horizontal movements. For the SeaTag-GEOs, temperature data were limited to 3–4 values a day with no depth records, forcing us to consider all depth layers in the search for matching temperature values, thus increasing the uncertainty of locations. Given these limitations, it is essential to conduct additional studies to assess the repeatability of results reported here and to work closely with manufacturers to push for further miniaturization of the tags and improvements in data retrieval capabilities.

In conclusion, despite the limitations imposed by currently available technologies, this study represents a significant step in understanding the migrations of this most enigmatic of species and illustrates the feasibility of revealing in even greater detail the migration routes and orientation cues eels use to complete their life cycle.

## Methods

### Pop-up archival satellite tags (PSATs)

A total of 38 silver eels were equipped with two different kinds of PSATs (Supplementary Table 1): 27 tags were X-tags from Microwave Telemetry (http://www.microwavetelemetry.com) and 11 were SeaTag-GEOs from Desert Star Company (http://desertstar.com/). Each X-tag measures 120 mm in length, has a maximum diameter of 32 mm and weighs 45 g in air. On board sensors collect and archive data on depth, water temperature and light every 2 min. X-tags were programmed to record 12-bit resolution measurements of light, temperature (range −4 °C to+40 °C, 0.23 °C accuracy) and pressure (range 0–1,296 m, 0.3–5 m resolution) and to store the records in the 64 Mb FLASH memory. At the end of each day (Universal Time Coordinates), the archived data for the previous 24 h is processed within the tag to build up a subset of the data (15-min intervals for temperature and depth, minimum and maximum light level and sunrise and sunset estimates) for transmission to the Argos low earth orbiting satellite system (http://www.argos-system.org/) after tag release. In case of premature death of the host or detachment of the tag from its host, the X-tags were programmed to initiate the pop-up procedure and transmit data after 7 consecutive days of constant depth readings (±3 m) with a 15-day delay following deployment (that is, the tag ignores constant pressure for the first 15 days). The SeaTag-GEOs are 132 mm in length, 13 mm in diameter for the main section and a weight 29 g in air. Their internal memory allowed to record temperature (−40 to +85 °C, 0.2 °C accuracy) and geomagnetic field values (3 axes) either three or four times a day during 3–4.5 months. Light sensors are also on board so day length and noon estimates are also transmitted. The SeaTag-GEOs were programmed to transmit both raw data and daily average for 2 months after the programmed dates. Unlike the X-tags, the SeaTag-GEOs have a solar battery and transmit their data continuously, that is, as soon as they are at the surface, satellites can pick up the data.

### Capture and eel tagging

All eels used in the experiments were wild silver eels caught while performing their downstream migration from fresh or brackish waters. They were all caught by commercial fishermen who used fyke nets and were kept for several days in appropriate basins before retrieval. To minimize the negative effects of drag caused by the external tags, eels were selected for tagging on the basis of their large size and body mass. In 2012 and 2013, the selected eels originated from NS and were caught and released at the same location. In 2012, several locations in NS were visited in order to find the largest eels. In 2014, based on the previous year's experiments and results, it was decided to use the largest eels that can be found in the entire species range: eels from the St. Lawrence system^31^. Indeed, the latter were on average 109 cm in total length (maximum of 120 cm) and 2.9 kg in body mass (maximum of 3.7 kg), whereas the largest eels that we found in NS reached a maximum of 93 cm in total length (85 cm on average) and 2.0 kg in body mass (1.4 kg on average; Supplementary Table 1). The eels from the St. Lawrence system were caught in the brackish estuary (Rivière Ouelle, 47.44°N, 70.03°W, Fig. 1) and transported by truck to the tagging and release location in Blandford, NS (Supplementary Table 1 and Fig. 1) at ca 860 km of driving from the capture location. It represents an aquatic shortcut of around 1,400 km for translocated eels, which consequently did not have to cross the lower estuary and the Gulf of St. Lawrence to reach the open ocean, thereby avoiding high predation^6^.

The tagging procedure (surgery and tag attachment method) was the same as previously used and detailed in Béguer-Pon *et al*.^6^ but for the last 2 years two attachment points instead of four were used. Furthermore, based on a recent study about the tag effect^30^, it was decided for the last year of experiment to attach the tag closer to the head of the eels (0.125 body length from the tip of the snout) instead of at their centre of mass (0.35 body length), in order to reduce the potential negative impact due to the drag of the tag.

For all years' experiments, tagged eels were released at the same time along with 12 non-tagged eels since swimming in schools can provide fish with a number of behavioural and ecological advantages, such as reduced predation risk or energy saving^32^. It is not really known whether or not silver eels swim in schools during their oceanic migration but there are reports indicating eels tend to aggregate in large groups during their seaward migration^33^. It was also observed that silver eels migrating down the St. Lawrence River show a synchrony in the time of their passage in the brackish estuary^5^,^34^, suggesting that eels could travel together during the marine phase of the migration. In 2012, eels were released in very shallow waters, directly from the beaches or docks at the tagging locations. In 2013 and 2014, eels were transported and released 5–10 km offshore, where the water depth is 30–50 m.

This study was carried out in strict accordance with the recommendations of the Canadian Council on Animal Care. The protocol was approved by the Animal Care Committee, Laval University (Permit Number 2011101-01) and Maurice-Lamontagne Institute, Fisheries and Oceans Canada (Permit Number 12-6C). All surgery was performed under acetyleugenol (220 p.p.m.) and all efforts were made to minimize suffering.

### Reconstructing the daily locations

*Geolocation of ‘pop-up'/detachment events.* All tags popped up earlier than the programmed dates. Except for two tags that were ingested by homeothermic fish, the reason for premature release could not be identified and could be various: failure in the attachment system, predation by ectothermic fishes or death of the eels. The release mechanism of X-tags was triggered by constant pressure during 7 consecutive days; they were actually drifting at the surface for 7 days before the first transmitting location was calculated by Argos system. Therefore, the first transmitting locations were not the locations where the tags detached and reached the surface. We thus inferred the location of their detachment using the temperature and light data collected at the surface. Sunset and sunrise estimates were used to calculate the longitude (with a 0.5° uncertainty), whereas the latitude was inferred from the surface water temperature (±1 °C). The package ‘oce' in R^35^,^36^ was used to calculate the longitude from sunrise and sunset. For all X-tags, sunset and sunrise estimates from the day of their detachment or following it could not be used as they were clearly erroneous. We used the maximum drifting distance observed during 5 days after the beginning of the transmission to increase the longitudinal search limits, as well as the directions of currents observed while the tags were drifting. As previously mentioned, data transmitted by the SeaTag-GEOs can be received by satellites as soon as the tags are at the surface, leading to only a few hours of drift before detection in most cases. Their first transmitting Argos location may thus reflect the location where the tag reached the surface. However, for some SeaTag-GEOs it was noticed that reliable sunset and sunrise were provided 2–5 days before the first locations were calculated by the Argos system, indicating these tags were probably drifting at the surface during that period (no depth sensor on these tags) but the data were not transmitted right away (for unknown reasons). We thus used the same method as for the X-tags to infer the geolocation of the pop-up events.

Temperatures recorded by the tags at the surface were matched with strongly assimilated physical models: HYCOM for data of 2012 and 2013 experiments and the operational Mercator global ocean 1/12° analysis and forecast system for 2014 experiments as HYCOM had missing data during our 2014 tracking period.

HYCOM has 1/12° equatorial resolution and latitudinal resolution of 1/12° cos(lat) or ∼7 km for each variable at mid-latitudes. It has 40 coordinate surfaces in the vertical. The data assimilation is performed using the Navy Coupled Ocean Data Assimilation^37^ system with a model forecast as the first guess. Navy Coupled Ocean Data Assimilation assimilates available satellite altimeter observations (along the track obtained via the NAVOCEANO Altimeter Data Fusion Center), satellite and *in situ* sea surface temperatures as well as available *in situ* vertical temperature and salinity profiles from XBTs, ARGO floats and moored buoys.

The operational Mercator global ocean 1/12° analysis and forecast system uses the NEMO 3.1 (Nucleus for European Models of the Ocean) modelling system, coupled to the thermodynamic-dynamic sea ice model LIM2 (Louvain sea Ice Model 2). The ocean model has a horizontal resolution of 9 km at the equator, 7 km at Cape Hatteras (mid-latitudes) and 2 km towards the Ross and Weddell seas. The ocean model has 50 levels in the vertical with 1 m resolution at the surface decreasing to 450 m at the bottom, and 22 levels within the upper 100 m. The 3-hourly atmospheric fields forcing the ocean model are taken from the European Centre for Medium-Range Weather Forecasts Integrated Forecast System. This modelling system assimilates jointly satellite sea level anomaly (Jason2, Cryosat, Saral-Altika) and sea surface temperature (Reynolds AVHRR-AMSR 1/4°), and *in situ* profiles of temperature and salinity. A detailed description of the modelling system and the quality of its product can be found at http://www.myocean.eu/web/69-myocean-interactive-catalogue.php?option=com_csw&view=details&product_id=GLOBAL_ANALYSIS_FORECAST_PHYS_001_002. Salinity is reported using the Practical Salinity Scale.

We checked that the modelled sea surface temperature was compatible with the temperature observed by the tags during their free drifting stage. It should be noted that the modelled sea surface temperature represents the daily mean temperature of the top 1 m water column. However, the temperature sensor on the tag measures the water temperature at a depth of ∼5 cm while drifting at the surface.

For each daily reconstructed location, we calculated the distance between the minimum and maximum estimates of latitude and longitude. These distances provided a measure of the uncertainty around the daily reconstructed locations. The uncertainty around the estimated pop-up locations varied among tags and was on average 120±94 km in latitude (mean±s.d.; range: 9–288) and 77±37 km in longitude (range: 20–148; Supplementary Table 3).

*Geolocation of daily tracks.* Traditionally, light data are used to infer the longitude^9^ but as eels avoid the euphotic zone during daytime and the light sensors on the tags are not sensitive enough to record reliable sunset and sunrise estimates^10^, we developed another method similar to the one used in Westerberg *et al*.^12^. According to the data recorded by the two kinds of tags, we used the bathymetry, DVM behaviour and temperatures at specific depths to infer the geolocation of eels equipped with X-tags and the temperature (point records, not daily averages) and the geomagnetic field total intensity for eels equipped with SeaTag-GEOs.

In some cases, the depths recorded by X-tags were assumed to be the bottom, as a constant value for several hours and days was observed. This was the case for several tags for only a few days following their release. The daily geolocation in these cases were inferred by matching the observed water depth and associated temperature to the 30 arc-second GEBCO bathymetry and the results from the operational ocean circulation model assuming the maximum distance eels could have travelled in one day in any direction to be 60 km.

When clear DVM patterns were observed, we estimated the sunrise and sunset times from the vertical profiles using the statistical R package ‘breakpoints', which implements the cross-entropy method^38^. This method is based on a stochastic optimization technique to estimate both the number and their corresponding locations of break-points in biological sequences of continuous and discrete measurements. Estimating sunrise and sunset from vertical profiles of eels was used in Westerberg *et al*.^12^ to calculate the longitude. Furthermore, Chow *et al*.^10^ tracked several Japanese eels (*A. japonica*) using ultrasonic transmitters and determined that eels started descending 55 min before sunrise (±10 min) and started ascending at sunset (±2 min). Considering the individual and daily variability described in Chow *et al*.^10^ and considering our sampling rate (15 min versus 2 min in the study that used acoustic tags) we decided to apply a 15-min uncertainty around the sunrise and sunset estimates. This leads to an average of±1.2° uncertainty in longitude (that is, around 400 km). The possible daily locations of eels equipped with X-tags were then further constrained by searching the modelled temperature field from the operational ocean circulation model within the range of mean±s.d. of observed temperature at the maximum depth that was reached by the eel each day and the temperature in the depth layer 0–200 m.

For eels equipped with SeaTag-GEOs, as there was no depth record, we matched the temperatures recorded by the tags with temperatures from the HYCOM model for all depth layers between the surface and 800 m. The geomagnetic field total intensity data (Gnt) recorded by the SeaTags-GEOs were matched with the modelled values from the International Geomagnetic Reference Field–IRGF- (www.ngdc.noaa.gov/IAGA/vmod/irgf.html). The real-time tracking data (while the tags were drifting) were used to calibrate the geomagnetic field values. The discrepancy between the Gnt recorded by the tags at the surface and the modelled data from IGRF was on average of 800 nT (range: 92–4,000 nT). For each tag, the calculated standard deviation of the discrepancy between the tag and the model was used as a measure of uncertainty around the Gnt value recorded by the tag for constraining the geolocation. Some Gnt values were obviously erroneous and thus not taken into account for the constraint. Although the constraint using temperature generally led to latitudinal error estimates, the Gnt constraint led to oblique strips because of the natural gradient of this environmental data.

Following the constraints from the environmental data, the inferred locations were finally constrained using both backward and forward in time tracking procedures with a maximum daily travel speed we assume the eels could have gone in any direction (60 km per day). No hypothesis about preferred directions were made.

### Methodological limitations of the PSAT technology

The placing of PSAT on a relatively small marine species such as eel may adversely affect behaviour and produce distorted patterns of movement and erroneous interpretations of migratory behaviour, as observed with other species^39^. Several laboratory studies have shown that PSATs increase drag and can significantly impair the swimming performance of relatively small eels^28^,^29^,^30^. The eels tagged in 2013 were about half the body mass of the eels tagged in 2014. The ground migratory speeds between the edge of Scotian Shelf and the open waters at the exit of the Laurentian Channel was 2.2–5 times slower for the smallest eels compared with the largest eels, potentially reflecting increased drag from the PSAT affecting the smallest eels. The pop-up locations on the western part of the Scotian Shelf could also reflect the difficulty of eels to swim against the main westward current. The potential impact of carrying a PSAT on vertical migratory behaviour is unknown. As the DVM was also exhibited by eels tagged with internal acoustic tags^10^, the PSAT is not responsible for this behaviour. However, it remains unknown if the maximum and minimal depths at which eels swim could be affected by the external tag.

Another limitation of using PSATs comes from the data recorded by the tags and our ability to reconstruct the migratory paths. The X-tags record depth, temperature and light every 2 min but these data cannot be assessed until the tags are physically retrieved, which is just about impossible in our study area because of its vastness. A subset of data is transmitted to satellites: depth and temperature at 15-min intervals, minimal and maximum daily light levels and sunset and sunrise estimates. We used the depth data to infer the longitude, as sunset and sunrise estimates from light sensors were not available. Because of the data sampling rate and the individual and daily variability of DVM observed in another study^10^, the uncertainty in longitude estimate was around 1.2° (around 400 km). This uncertainty could be reduced with a higher sampling rate and better correlations between migration depth and light intensity in our study area. The latitudes were inferred using the temperature recorded at specific depths by X-tags. The uncertainty of the reconstructed latitudes thus comes from the accuracy of the recorded data and from the resolution and accuracy of the operational ocean circulation models used to compare with the archival data. In this study, the ocean models have horizontal resolutions of around 7–9 km, defining thus the minimal uncertainty of the reconstructed path and preventing assessment of finer horizontal movements. For the SeaTag-GEOs, temperature data were limited to 3–4 values a day with no depth records, forcing us to consider all depth layers in the search for matching values, thus increasing the uncertainty of locations. These tags record geomagnetic field total intensity values that allow us to constraint location in oblique strips. We noticed various issues with the geomagnetic data: we had to calibrate the values using real-time tracking data (while the tags were drifting) and high discrepancies between recorded data and the modelled data from the International Geomagnetic Reference Field (up to 4,000 nT) were noted. Furthermore, some of recorded geomagnetic values were obviously erroneous (for instance leading to inferred locations at several thousands of kilometres) and had to be discarded from the analysis. The overall uncertainty of reconstructed trajectories was higher in the case of SeaTag-GEOs compared with X-tags. X-tags have a higher sampling rate, do record depth, have more reliable data and had a higher transmitting rate (97% versus 48%).

## Additional information

**How to cite this article:** Béguer-Pon, M. *et al*. Direct observations of American eels migrating across the continental shelf to the Sargasso Sea. *Nat. Commun.* 6:8705 doi: 10.1038/ncomms9705 (2015).

## Supplementary Material

Supplementary Information Supplementary Figures 1-7 and Supplementary Tables 1-3

## Figures and Tables

**Table 1:** Summary of observed migratory patterns of wild silver eels equipped with PSAT and released off Nova Scotia.

Area	Area name	*N* tags	*N* end of tracking	Vertical behaviour (X-tags only)	Travel speed (km per day)	General heading relative to the release site	Overall direction of currents
A	Scotian Shelf	*N*_total_=20 *N*_2012_=2 XT *N*_2013_=4 ST *N*_2014_=14 XT	*N*_total_=8; *N*_2012_=2 XT *N*_2013_=4 ST *N*_2014_=6 XT*	DVM towards bottom (*N*_2012-2014_=8), ‘erratic' dives in shallow waters (*N*_2012-2014_=4)	34 (20–57, *N*_all years_=11)	S-SSE (*N*_all years_=16)	W
							
B	Scotian Shelf Edge	*N*_total_=12; *N*_2013_=4 ST *N*_2014_=8 XT	*N*_total_=6; *N*_2013_=1 ST *N*_2014_=5 XT	Erratic, shallow waters (*N*_2014_=8)	57–53 (*N*_2014_=2 XT); remained on the edge between 4 and 26 days (N_2013–2014_ =11)	E (*N*_2013–2014_=7) Unknown for *N*_2013_–_2014_=5 (unresolved W/E movements) but N_2014_=3 ended W	W
							
C	Beyond exit of Laurentian Channel (Open Ocean)	*N*_total_=8†; *N*_2013_=4 ST *N*_2014_=4 XT	*N*_total_=6; *N*_2013_=3ST *N*_2014_=3XT*	Erratic dives in shallow waters followed by DVM more or less discernable (*N*_2014_=2)	10–17 (*N*_2013_=3 ST) and 38–50 (*N*_2014_=2 XT)	SE	E
							
D	Gulf Stream	*N*_total_=2; *N*_2013_=1 ST *N*_2014_=1 XT	*N*_total_=1; *N*_2013_=1ST	DVM (*N*_2014_=1)	46 (*N*_2013_=1ST) and 52 (*N*_2014_=1 XT)	S	E
							
E	Sargasso Sea	N_total_=1; N_2014_=1 XT	N_total_=1; N_2014_=1XT	DVM (N_2014_=1)	49	S	W and then N

DVM, diel vertical migration; PSAT, pop-up satellite archival tag; ST, SeaTag-GEOs; XT, X-tags; S, South; SE, Southeast; SSE, South-southeast; W, West; E, East; N, North.

^*^One eel predated.

^†^One eel predated and one went beyond the Scotian Shelf slope but came back over the edge.
