# Supplementary material for: Direct observations of American eels migrating across the continental shelf to the Sargasso Sea
Source: Nat Commun. 2015 Oct 27;6:8705. doi: 10.1038/ncomms9705 (PMC4918406; doi:10.1038/ncomms9705)
Supplement: Supplementary Information — Supplementary Figures 1-7 and Supplementary Tables 1-3 [file ncomms9705-s1.pdf]

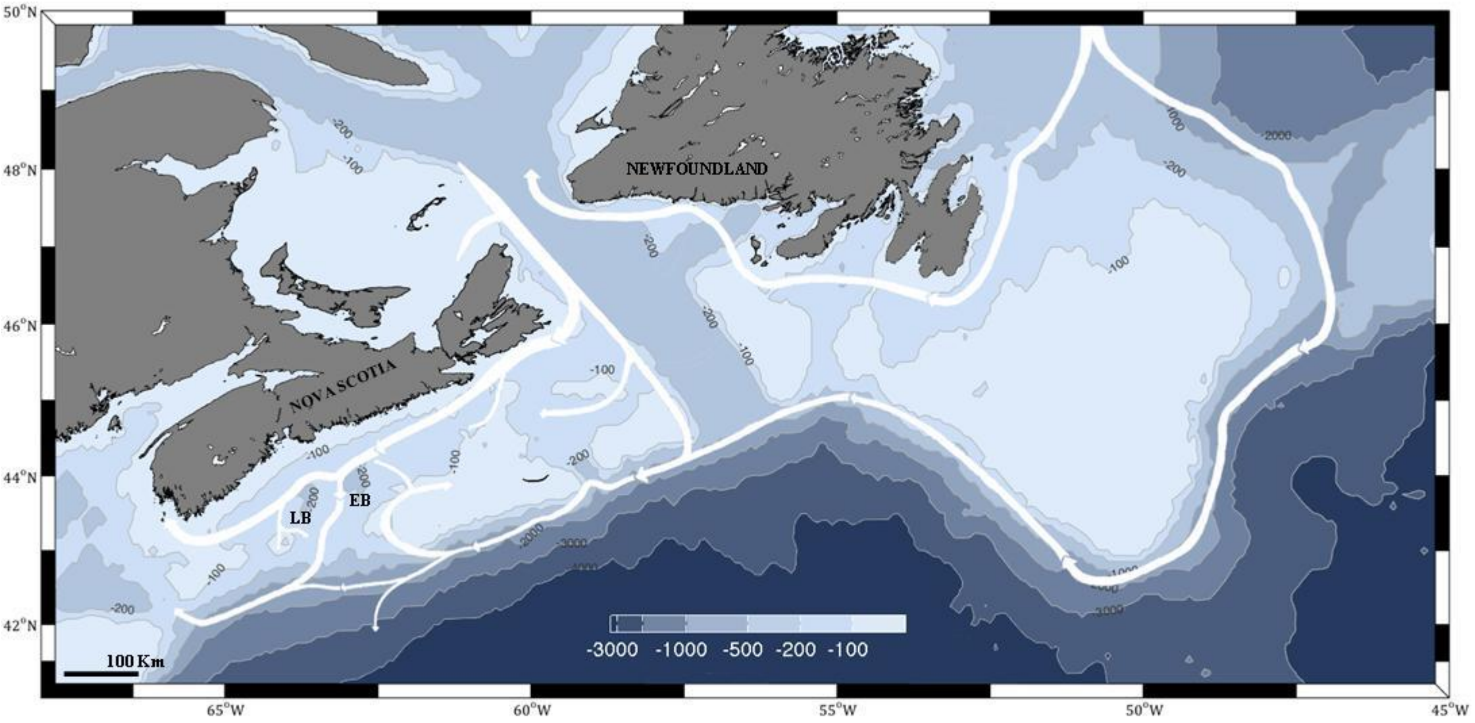

Supplementary Fig. 1. Schematic representation of the bathymetry and general surface circulation over the Scotian Shelf and the Grand Banks of Newfoundland. Figure from Fisheries and Oceans Canada, adapted by M. Dever (Dept. of Oceanography, Dalhousie University). The LaHave (LB) and Emerald (EB) Basins are located in the middle of Scotian Shelf with water depth >200 m.

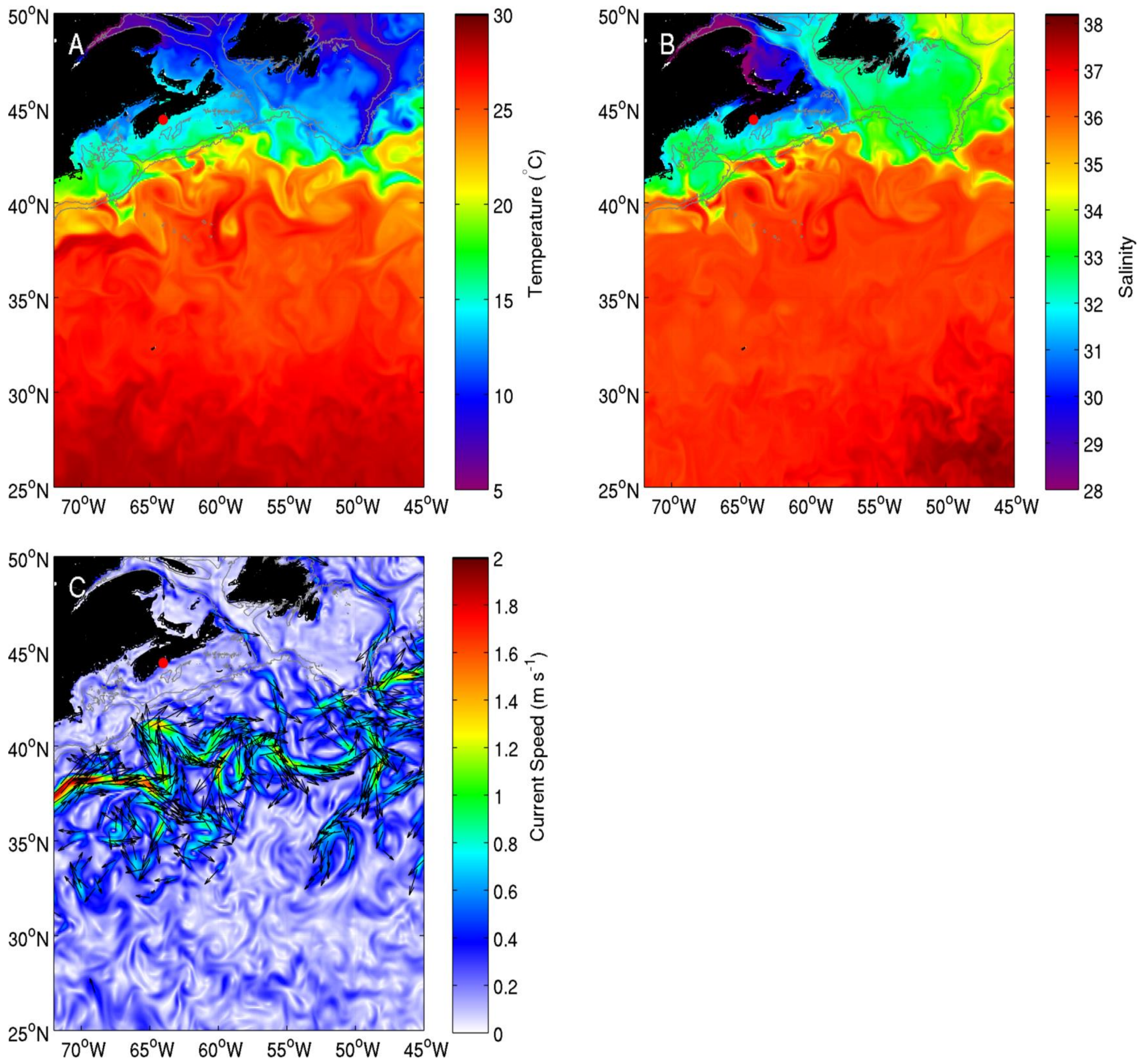

Supplementary Fig. 2. Snapshot of temperature, salinity and currents at surface on October 15, 2014. Data from operational Mercator global ocean 1/12° analysis and forecast system. Current directions for current speeds > 0.4 m s^{-1} are indicated by black arrows.

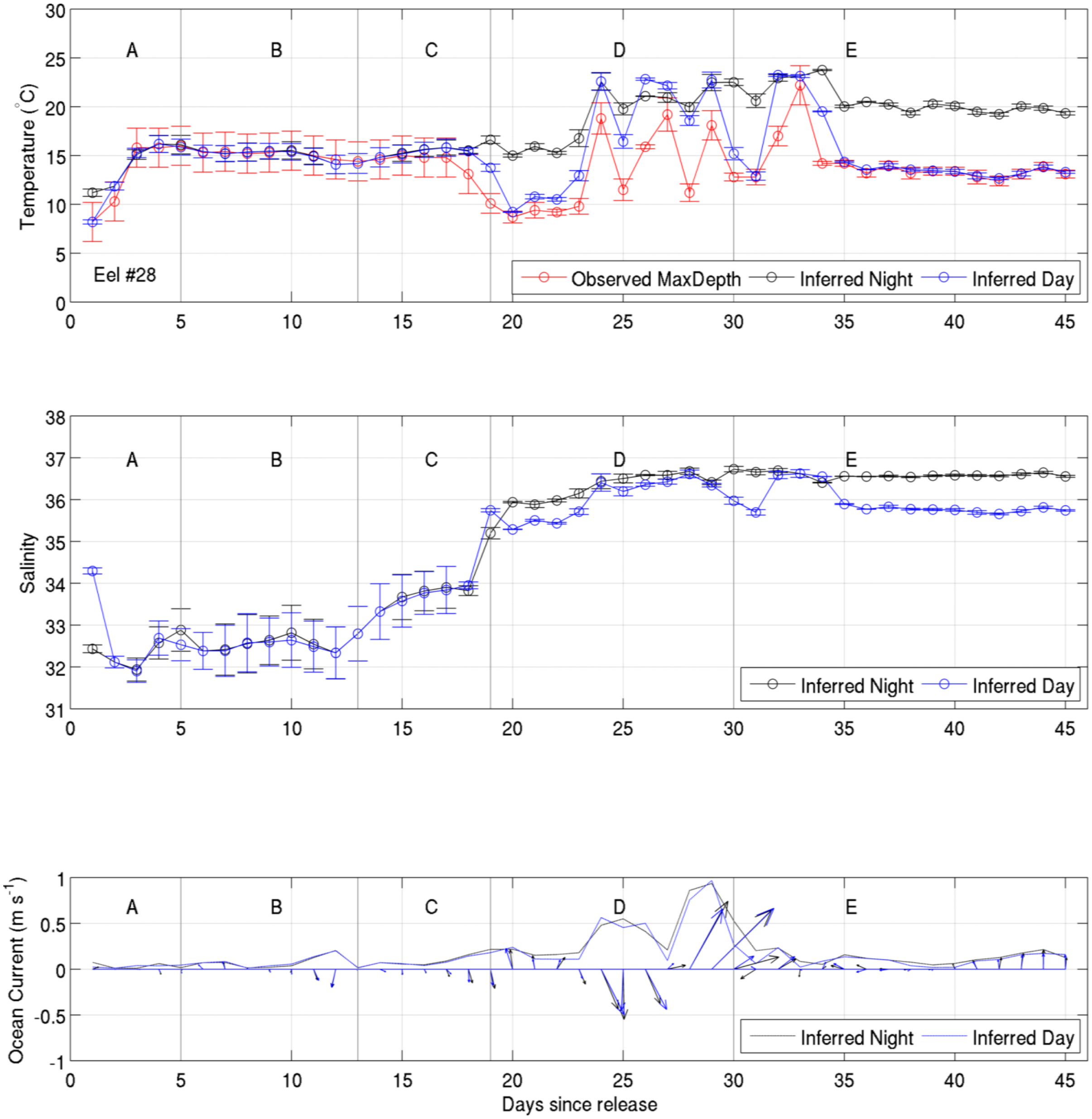

Supplementary Fig. 3. Temperature, salinity and current experienced by tagged eel #28 (X-tag #141105) along its reconstructed path from the coast to the northern limit of the spawning site in the Sargasso Sea. The red line represents the temperature observed near the daily maximum swimming depth. The black lines represent the inferred oceanic conditions experienced at night, i.e. mainly in shallow waters, whereas the blue lines are the inferred oceanic conditions experienced during daytime, i.e. in deeper waters. The error bars represent the standard deviation around the mean. Note that the greater standard deviation around the mean salinity in area B (see Fig. 1) comes from the high uncertainty of the reconstructed path in this area.

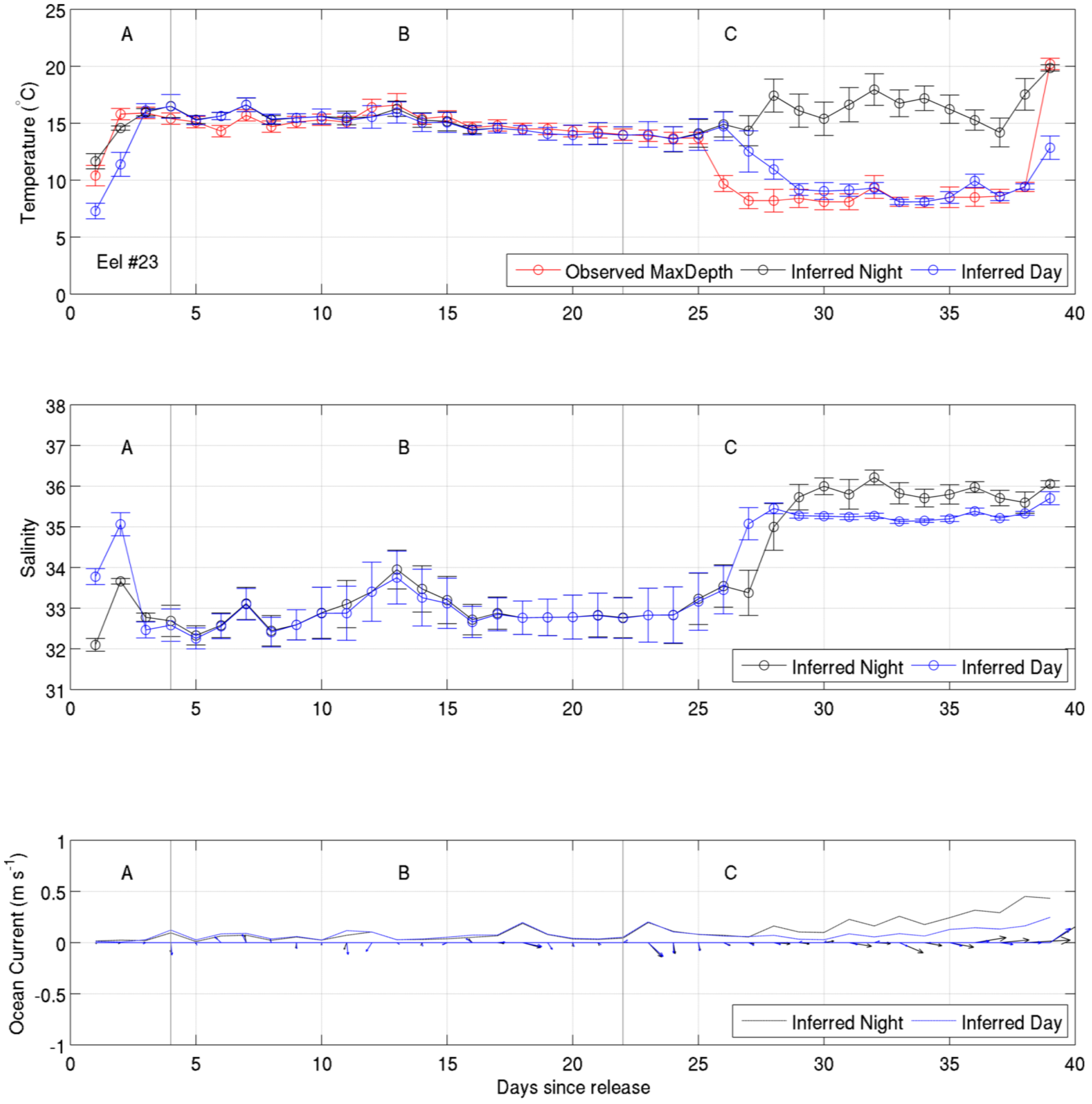

Supplementary Fig. 4. Same as Supplementary Fig. 3, but for tagged eel #23.

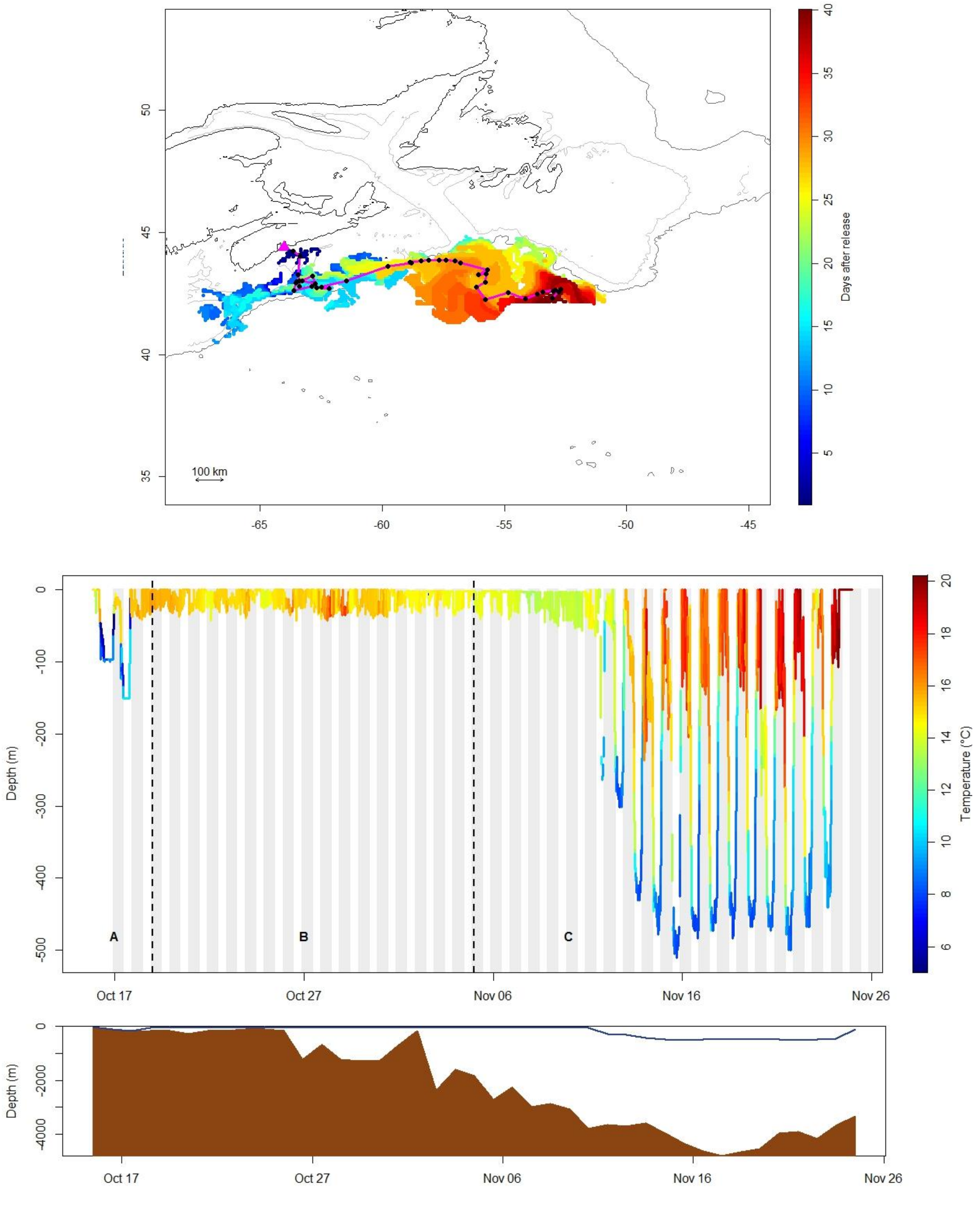

Supplementary Fig. 5. Horizontal and vertical behaviour of the tagged eel # 23 (X-tag #141112). Upper panel: reconstructed path from release site (magenta triangle). A color gradient is used to show the temporal dimension of the reconstructed trajectory (each day is represented by a color). The black dots are the mean reconstructed daily

locations and the magenta line is the corresponding mean trajectory. Lower panel: depth profile with temperature superimposed (color gradient). The letters A, B and C represents the geographical area of the eel: Scotian Shelf, Edge of Scotian Shelf and open ocean at the exit of the Laurentian Channel, respectively (see Fig. 1). The bottom panel shows the observed daily maximum eel depth (dark blue line) and the corresponding bottom depth at the daily reconstructed location (brown shaded curve).

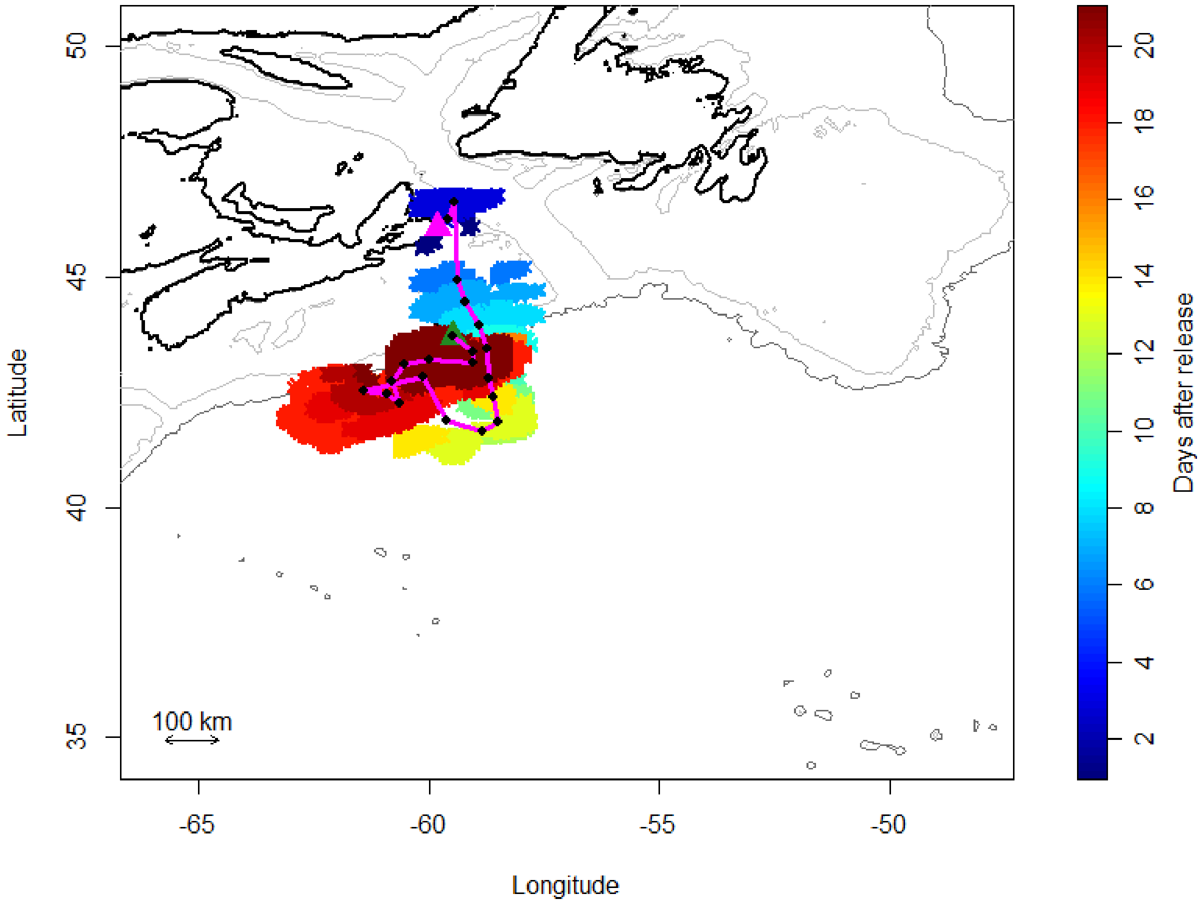

Supplementary Fig. 6. Reconstructed path of tagged eel #10 (SeaTag GEO #212), released on October 18, 2013. The magenta and green triangles represent the release and first transmitting locations, respectively. The 200 (light grey) and 2000 m (darker grey) isobaths are shown.

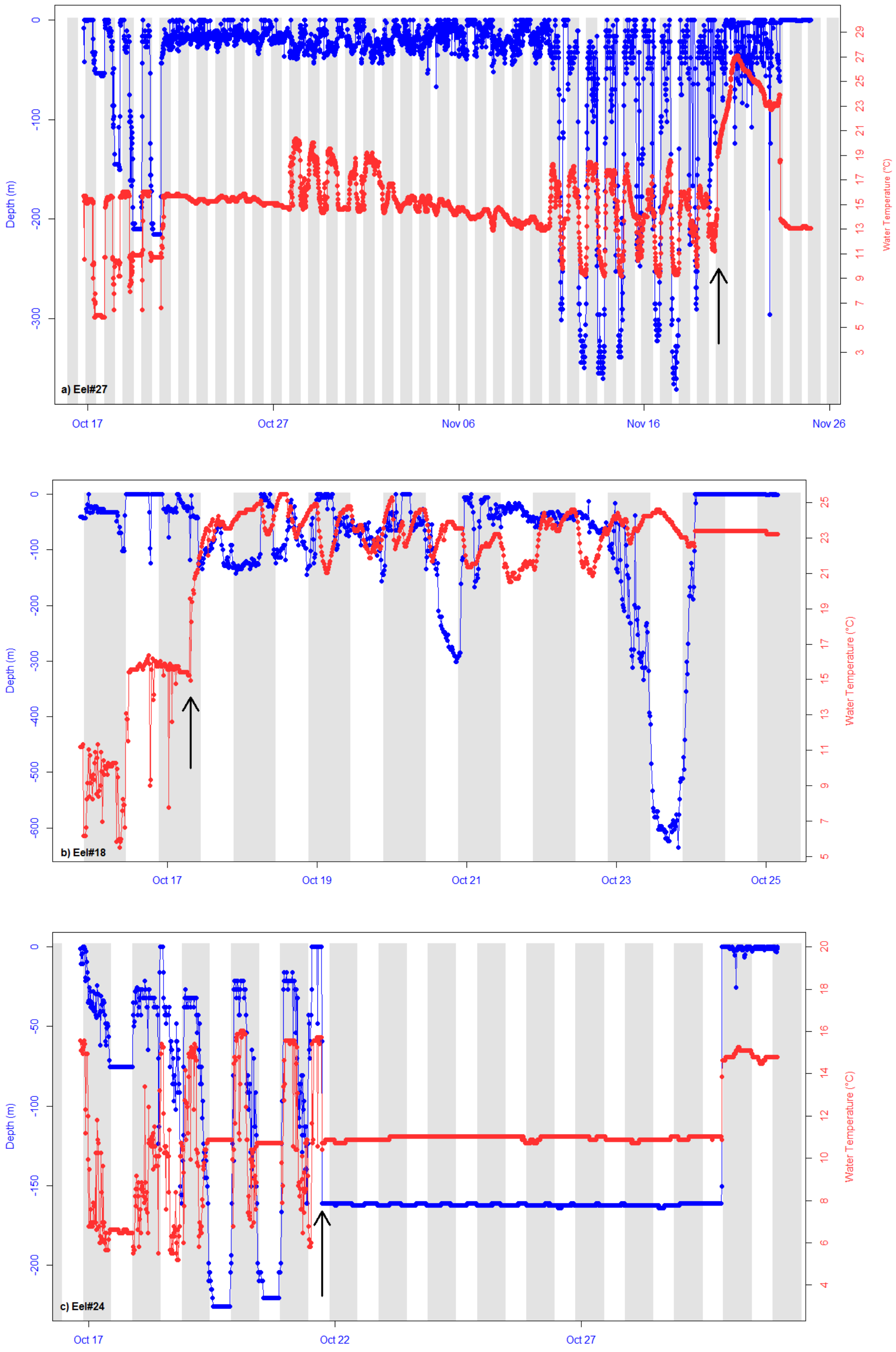

a) Eel#27
Depth (m)
Water Temperature (°C)
Oct 17
Oct 27
Nov 06
Nov 16
Nov 26
b) Eel#18
Depth (m)
Water Temperature (°C)
Oct 17
Oct 19
Oct 21
Oct 23
Oct 25
c) Eel#24
Depth (m)
Water Temperature (°C)
Oct 17
Oct 22
Oct 27

Supplementary Fig.7. Vertical profiles of three eels equipped with X-tags in 2014 showing predation events. Both Eel # 27 and Eel #18 were ingested by homeothermic fishes as demonstrated by the sudden increase in temperature (indicated by the black arrows) that cannot be related to water temperature in the study area in October-November. The grey bars represent night periods while the white bars represent daytime periods. Using the method developed by Béguer-Pon et al. (2011), the predator of eel #27 was identified as most likely a bluefin tuna and predator of eel #18 as a porbeagle shark. The depth reached by eel #27 before the predation event indicates that the predation occurred beyond the Scotian Shelf (39 days after its release from the coast) while eel #18 was predated 1.5 days after its release over the Scotian Shelf. In panel c, the black arrow shows a sudden descent of eel #24 toward the bottom, 4.5 days after the eel release, which could indicate a predation by a cold-gutted fish (since no increase of temperature is observed). The eel (or tag only) then remained on the bottom for 8 days before suddenly reaching the surface where the tag started to transmit its data 7 days later. Similar vertical behaviours that suggest predation of tagged eels by ectothermic fish a couple of days after their release were observed for 7 other eels equipped with X-tags (there is no depth sensor in SeaTag GEO): eel #1 and #2 released in 2012, eel #13 released in 2013, eels #15, #16, #17 and #19 released in 2014.

Supplementary Table 1. Capture, tagging, release and transmitting data for the 38 eels fitted with PSAT in falls 2012, 2013 and 2014

#PSAT	PSAT Type and ID	Lt (mm)	W (kg)	Release date	Release Location	Lat (°N)	Lon (°W)	Programmed release date	Date of first transmiting	Number of days after release	Lat (°N) First transmitting	Lon (°W) First transmitting	Great-circle distance between release and first transmitting position (km)	% data transmitted
1	X-Tag-110626	924	1.8	15/10/2012	Lower West Jeddore, NS	44.72	-63.01	15/03/2013	30/10/2012	15	44.28	-62.11	87	98
2	X-Tag-110619	930	1.9	15/10/2012	Lower West Jeddore, NS	44.72	-63.01	15/03/2013	31/10/2012	16	43.81	-64.24	140	100
3	X-Tag-120858	888	1.8	15/10/2012	Lower West Jeddore, NS	44.72	-63.01	15/03/2013	04/11/2012	20	44.65	-63.42	33	56
	X-Tag-110622	925	2.0	15/10/2012	Lower West Jeddore, NS	44.72	-63.01	15/03/2013						
	X-Tag-120860	932	1.9	15/10/2012	Lower West Jeddore, NS	44.72	-63.01	15/03/2013						
	X-Tag-120859	878	1.9	15/10/2012	Lower West Jeddore, NS	44.72	-63.01	15/03/2013						
	X-Tag-110623	810	1.4	20/10/2012	Mira Bay, NS	46.07	-59.88	15/03/2013						
	X-Tag-110625	818	1.1	20/10/2012	Mira Bay, NS	46.07	-59.88	15/03/2013						
	X-Tag-120861	795	1.1	20/10/2012	Mira Bay, NS	46.07	-59.88	15/03/2013						
	X-Tag-110621	826	1.1	20/10/2012	Mira Bay, NS	46.07	-59.88	15/03/2013						
4	SeaTagGEO-210	808	1.1	17/09/2013	Mira Bay, NS	46.07	-59.75	02/02/2014	09/10/2013	21.9	46.13	-59.87	11.4	98
5	SeaTagGEO-216	843	1.3	17/09/2013	Mira Bay, NS	46.07	-59.75	05/03/2014	18/09/2013	0.7	46.01	-59.89	13.7	
6	SeaTagGEO-218	791	1.1	17/09/2013	Mira Bay, NS	46.07	-59.75	30/12/2013	30/09/2013	12.7	44.72	-59.88	150.4	38
7	SeaTagGEO-217	877	1.6	17/09/2013	Mira Bay, NS	46.07	-59.75	30/12/2013	16/11/2013	59.7	41.81	-52.48	750.3	9
	SeaTagGEO-214	869	1.2	17/09/2013	Mira Bay, NS	46.07	-59.75	05/03/2014						
8	SeaTagGEO-209	828	1.1	18/10/2013	Mira Bay, NS	46.08	-59.81	05/03/2014	16/11/2013	28.8	46.12	-59.77	5.3	67
9	SeaTagGEO-211	838	1.2	18/10/2013	Mira Bay, NS	46.08	-59.81	30/01/2014	16/11/2013	28.7	45.75	-59.84	36.4	7
10	SeaTagGEO-212	836	1.2	18/10/2013	Mira Bay, NS	46.08	-59.81	30/01/2014	12/11/2013	24.7	43.71	-59.47	264.8	59
11	SeaTagGEO-215	870	1.2	18/10/2013	Mira Bay, NS	46.08	-59.81	05/03/2014	25/11/2013	37.8	42.90	-53.43	616.6	24
12	SeaTagGEO-213	829	1.3	18/10/2013	Mira Bay, NS	46.08	-59.81	05/03/2014	25/11/2013	37.9	41.54	-52.73	759.1	33
13	X-Tag-110624	862	1.3	18/10/2013	Mira Bay, NS	46.08	-59.81	15/01/2014	29/10/2013	10.3	45.85	-59.64	28.6	100
	X-Tag-120857	840	1.5	18/10/2013	Mira Bay, NS	46.08	-59.81	15/01/2014						

Supplementary Table 1. (continued)

PSAT ID	PSAT Type and ID	Lt (mm)	W (kg)	Release date	Release Location	Lat (°N)	Lon (°W)	Programmed release date	Date of first transmiting	Number of days after release	Lat (°N) First transmitting	Lon (°W) First transmitting	Great-circle distance betwen release and first transmitting position (km)	% data transmitted
14	SeaTagGEO-209b	1070	2.8	15/10/2014	Off Blandford, NS	44.39	-64.03	05/01/2015	19/10/2014	3.7	44.08	-64.00	37	100
15	X-Tag-141113	1170	3.7	15/10/2014	Off Blandford, NS	44.39	-64.03	15/03/2015	30/10/2014	14.4	43.02	-65.04	174	100
16	X-Tag-141106	1100	3.2	15/10/2014	Off Blandford, NS	44.39	-64.03	15/01/2015	30/10/2014	14.9	43.15	-64.64	146	100
17	X-Tag-141107	1110	2.6	15/10/2014	Off Blandford, NS	44.39	-64.03	15/01/2015	31/10/2014	15.2	43.39	-64.32	113	100
18	X-Tag-141108	1020	2.4	15/10/2014	Off Blandford, NS	44.39	-64.03	15/01/2015	31/10/2014	15.3	39.56	-67.07	602	100
19	X-Tag-141114	1060	2.8	15/10/2014	Off Blandford, NS	44.39	-64.03	15/03/2015	01/11/2014	16.1	43.09	-63.21	158	100
20	X-Tag-141109	1110	3.2	15/10/2014	Off Blandford, NS	44.39	-64.03	15/01/2015	05/11/2014	20.9	42.71	-65.72	231	100
21	X-Tag-141116	1130	3.5	15/10/2014	Off Blandford, NS	44.39	-64.03	15/03/2015	05/11/2014	20.9	42.82	-65.15	194	100
22	X-Tag-141115	1040	2.7	15/10/2014	Off Blandford, NS	44.39	-64.03	15/03/2015	08/11/2014	23.4	43.38	-60.04	340	100
23	X-Tag-141112	1120	2.9	15/10/2014	Off Blandford, NS	44.39	-64.03	15/03/2015	01/12/2014	46.7	41.30	-48.89	1275	98
24	X-Tag-141119	1060	2.8	16/10/2014	Off Blandford, NS	44.39	-64.03	15/03/2015	07/11/2014	21.5	43.58	-63.47	99.8	100
25	X-Tag-141111	980	2.2	16/10/2014	Off Blandford, NS	44.39	-64.03	15/01/2015	09/11/2014	23.7	42.58	-61.62	280	100
26	X-Tag-141110	1110	2.6	16/10/2014	Off Blandford, NS	44.39	-64.03	15/01/2015	19/11/2014	34.1	42.11	-64.48	255	99
27	X-Tag-141118	1200	3.0	16/10/2014	Off Blandford, NS	44.39	-64.03	15/03/2015	30/11/2014	44.7	42.88	-59.81	375	98
28	X-Tag-141105	1130	2.8	16/10/2014	Off Blandford, NS	44.39	-64.03	15/01/2015	08/12/2014	52.7	32.37	-58.26	1430	99
	X-Tag-141117	1060	2.9	16/10/2014	Off Blandford, NS	44.39	-64.03	15/03/2015						

Supplementary Table 2. Summary analyses of the archival data recorded by the tags while attached to the eels (*Number of days of activity before extended final period at the bottom or the surface, or predation event; Mean temperature is not provided for SeaTag GEO due to significant gaps in transmitted data.)

# PSAT	PSAT Type and ID	Nb days of activity *	Great-circle distance from release to estimated final surface (if available) (Km)	Travelled distance calculated using the mean reconstructed path (Km)	Global Bearing (°)	Extended period at bottom	Vertical Behaviour	Mean Depth (m)	Max Depth (m)	Nb of dives per day (Mean±Sd)	Proportion of Time spent within the first 50 m (Mean±Sd)	Range of vertical movements (Mean±Sd (Max)) (in m)	Mean Temperature experienced (°C)	Temperature range (°C)
1	X-Tag-110626	8	77 to 205	77 to 205	160-198	13.3 days	Multiples dives in shallow waters	-5	-97	7.3 ± 4.0	1	17 ± 10 (97)	15 ± 1.6	8.2 - 15.8
2	X-Tag-110619	3	86	86	190	6.2 days	DVM bottom	-35	-134	7.3 ± 4.0	0.81 ± 0.28	35 ± 27 (129)	10.3 ± 3.1	5.7 - 18.4
3	X-Tag-120858	0.5	-	-	-	-	Surface only	0	56	1	1	0	-	-
6	SeaTagGEO-218	12.7	151	-	184	*NA*	*NA*	*NA*	*NA*	*NA*	*NA*	*NA*	-	14.9-18.0
7	SeaTagGEO-217	57.2	589	1287	126	*NA*	*NA*	*NA*	*NA*	*NA*	*NA*	*NA*	-	13.4-17.3
9	SeaTagGEO-211	28.7	36.4	-	191	*NA*	*NA*	*NA*	*NA*	*NA*	*NA*	*NA*	-	5.9-18.9
10	SeaTagGEO-212	24.7	263	-	174	*NA*	*NA*	*NA*	*NA*	*NA*	*NA*	*NA*	-	2.6-21.6
11	SeaTagGEO-215	31.8	651	-	136	*NA*	*NA*	*NA*	*NA*	*NA*	*NA*	*NA*	-	11.0-13.8
12	SeaTagGEO-213	37.1	555	1744	118	*NA*	*NA*	*NA*	*NA*	*NA*	*NA*	*NA*	-	2.5-17.8
13	X-Tag-110624	1.5	29	29	152	9.0 days	1 DVM	-27	-54	1	0.47	7 ± 1 (8)	10.1 ± 2.8	7.1-13.2
14	SeaTagGEO-209b	3.7	37	-	-	*NA*		*NA*	*NA*		*NA*	*NA*	-	13.3-15.9
16	X-Tag-141106	0.7	41 ± 9	41	155-186	6.9 days	Reversed DVM	-30	-129	9	0.79	39 ± 33 (129)	11.3 ± 3.2	5.8-15.4
15	X-Tag-141113	0.8	42 ± 3	42	148-200	6.6 days	Descending	-44	-110	7	0.57	26 ± 19 (81)	10.2 ± 2.8	5.7-16.4

Supplementary Table 2 (continued)

#PSAT	PSAT Type and ID	Nb days of activity*	Great-circle distance from release to estimated final surface (if available) (Km)	Travelled distance calculated using the mean reconstructed path (Km)	Global Bearing (°)	Extended Period at bottom	Vertical Behaviour	Mean Depth (m)	Max Depth (m)	Nb of dives per day (Mean±Sd)	Proportion of Time spent within the first 50 m (Mean±Sd)	Range of vertical movements (Mean±Sd (Max)) (in m)	Mean Temperature experienced (°C)	Temperature range (°C)
17	X-Tag-141107	0.8	41 ± 3	41	124-186	7.3 days	Descending	-37	-124	8	0.83	35 ± 28 (116)	10.9 ± 2.3	5.8-16.2
18	X-Tag-141108	1.5 *(then predated)*				No	Reversed DVM	-23	-124	3	0.9	47 ± 38 (124)	12.7 ± 3.4	5.5-16.4
24	X-Tag-141119	4.9	111 ± 45	111	111-177	8.1 days	DVM	-87	-226	7.8 ± 3.2	0.46 ± 0.19	40 ± 37 (205)	10.2 ± 3.1	5.2-16
19	X-Tag-141114	7.0	111 ± 45	111	111-177	1.8 days	DVM for 2 days	-91	-215	10.1 ± 6	0.21 ± 0.24	38 ± 26 (172)	9.2 ± 2.9	4.9-16.5
21	X-Tag-141116	13.7	281 ± 80	680	201-234	No	Remained mostly in shallow waters (<30m)	-9	-145	11.3 ± 5	0.97 ± 0.05	22 ± 23 (140)	15.1 ± 1.3	5.7-16.4
20	X-Tag-141109	13.8	354 ± 64	744	200-222	No	Reversed and unclear DVM	-11	-97	8.8 ± 3	0.99 ± 0.04	18 ± 11 (94)	15.2 ± 1.1	5.7-16.7
22	X-Tag-141115	16.1	232 ± 48	600	182-215	No	DVM for the first 4 days	-30	-242	9.1 ± 5.6	0.84 ± 0.25	34 ± 39 (204)	15.4 ± 2.7	5.7-18.6
25	X-Tag-141111	17.1	230 ± 67	649	145-177	No	Clear DVM for the first 3.5 days	-32	-194	10.9 ± 4.4	0.89 ± 0.21	26 ± 23 (188)	15.2 ± 3	5.7-19.9
26	X-Tag-141110	26.9	232 ± 48	982	182-215	No	No DVM, remained in shallow waters	-16	-156	12.1 ± 3.4	0.99 ± 0.01	23 ± 14 (156)	14.8 ± 1.3	5.5-17.9

27	X-Tag-141118	34.1 *(then predated)*				No	DVM at the end, more or less marked	-50	-371	12.1 ± 6.1	0.82 ± 0.24	50 ± 81 (371)	14.6 ± 2.5	5.7-20.4
23	X-Tag-141112	39.4	918 ± 22	1750	98-103	No	Very clear and marked DVM for the last 2 weeks	-82	-511	14.6 ± 4.0	0.73 ± 0.36	53 ± 85 (511)	14.2 ± 2.6	5.0-20.2
28	X-Tag-141105	45.5	1570 ± 43	2426	158-161	No	Very marked DVM the last 10 days	-144	-699	12.9 ± 4.2	0.58 ± 0.36	68 ± 95 (678)	16.8 ± 4.2	5.7-25.1

Supplementary Table 3. Uncertainty of the geolocations inferred from environmental data

			Average uncertainty of the daily reconstructed geolocation		Uncertainty of the reconstructed pop-up location	
**#PSAT**	**PSAT Type and ID**	**Nb days of activity**	**in latitude (km, Mean±SD (Max))**	**in longitude (km, Mean±SD (Max))**	**in latitude (km)**	**in longitude (km)**
1	X-Tag-110626	8	103 ± 70 (205)	175 ± 97 (306)	133	84
2	X-Tag-110619	3	27 ± 32 (62)	40 ± 36 (70)	18	51
7	SeaTagGEO-217	57.2	348 ± 172 (621)	540 ± 282 (988)	111	72
10	SeaTagGEO-212	24.7	118 ± 62 (250)	186 ± 107 (433)	radius <500 m (Argos)	
11	SeaTagGEO-215	31.8	82 ± 67 (232)	117 ± 102 (353)	129	148
12	SeaTagGEO-213	37.1	277 ± 184 (615)	436 ± 303 (972)	27	53
16	X-Tag-141106	0.7	9	20	9	20
15	X-Tag-141113	0.8	9	33	9	33
17	X-Tag-141107	0.8	9	40	9	40
24	X-Tag-141119	4.9	111± 60 (175)	87 ± 47 (155)	167	80
19	X-Tag-141114	7.0	111± 26 (129)	210 ± 180 (337)	93	60
21	X-Tag-141116	13.7	226 ± 162 (445)	285 ± 185 (508)	288	110
20	X-Tag-141109	13.8	201 ± 102 (297)	330 ± 173 (531)	269	118
22	X-Tag-141115	16.1	100 ± 48 (167)	230 ± 160 (471)	121	116
25	X-Tag-141111	17.1	161 ± 104 (306)	258 ± 198 (581)	250	69
26	X-Tag-141110	26.9	309 ± 152 (473)	545 ± 339 (1002)	213	123
23	X-Tag-141112	39.4	202 ± 95 (380)	513 ± 271 (822)	46	96
28	X-Tag-141105	45.5	107 ± 91 (278)	137 ± 110 (305)	148	40
